# Adjusting for treatment changes in epilepsy: causal analysis of randomised controlled trial survival outcomes in the presence of departure from randomised treatment

**DOI:** 10.1186/1745-6215-14-S1-O37

**Published:** 2013-11-29

**Authors:** Susanna Dodd, Paula Williamson, Ruwanthi Kolamunnage Dona, Marta Van der Hoek, Ian White

**Affiliations:** 1 University of Liverpool, Liverpool, UK; 2 MRC Biostatistics Unit, University of Cambridge, Cambridge, UK

## 

Treatment changes are typical in epilepsy because of the chronic nature of the disease and common problems with treatment, most typically inadequate seizure control and unacceptable adverse effects. Randomised trials in epilepsy often adopt a pragmatic approach to treatment changes, in order to cater for patient needs and mirror the real-life experience of patients. Trial patients may experience multiple treatment changes taking a variety of forms, including addition of alternative treatment(s), switching to other treatment(s) and complete withdrawal from all treatment.

Primary analysis of trial data is usually based on the principle of intention to treat (ITT), which ignores such treatment changes and thus avoids any selection bias that may be introduced by changes from randomised treatment. ITT however only allows estimation of the effectiveness of treatment, rather than the true efficacy (or causal effect) of treatment, which is of particular interest to patients and clinicians alike in this setting.

Methods such as per protocol or as treated analyses are commonly implemented to estimate causal effects, but are often biased because treatment changes are typically associated with prognostic factors. There exist alternative statistical methods to estimate causal effects of treatment whilst avoiding such bias, such as the rank preserving structural failure time model (RPSTFM) and inverse probability of censoring weighting (IPCW) models, but these methods are not well known and require certain assumptions. I will discuss the challenges of adjusting for treatment changes in epilepsy and demonstrate the RPSTFM and IPCW methods, discussing their advantages and disadvantages in this setting.

